# Balancing false positives and false negatives for the detection of differential expression in malignancies

**DOI:** 10.1038/sj.bjc.6602140

**Published:** 2004-08-31

**Authors:** F De Smet, Y Moreau, K Engelen, D Timmerman, I Vergote, B De Moor

**Affiliations:** 1 Department of Electrical Engineering, ESAT-SCD, K.U.Leuven, Kasteelpark Arenberg 10, 3001 Leuven-Heverlee, Belgium; 2 Department of Obstetrics and Gynecology, University Hospitals, K.U.Leuven, Herestraat 49, 3000 Leuven, Belgium

**Keywords:** microarray, differential expression, multiple testing, ROC curve, acute leukaemia

## Abstract

A basic problem of microarray data analysis is to identify genes whose expression is affected by the distinction between malignancies with different properties. These genes are said to be differentially expressed. Differential expression can be detected by selecting the genes with *P*-values (derived using an appropriate hypothesis test) below a certain rejection level. This selection, however, is not possible without accepting some false positives and negatives since the two sets of *P*-values, associated with the genes whose expression is and is not affected by the distinction between the different malignancies, overlap. We describe a procedure for the study of differential expression in microarray data based on receiver-operating characteristic curves. This approach can be useful to select a rejection level that balances the number of false positives and negatives and to assess the degree of overlap between the two sets of *P*-values. Since this degree of overlap characterises the balance that can be reached between the number of false positives and negatives, this quantity can be seen as a quality measure of microarray data with respect to the detection of differential expression. As an example, we apply our method to data sets studying acute leukaemia.

Microarrays allow for the simultaneous measurement of expression levels of thousands of genes in tissues originating from different classes of malignancies (e.g., normal and malignant tissues (Alon *et al*, 1999); tumours that are and are not sensitive to chemotherapy (Kihara *et al*, 2001); tumours with good and poor prognosis (van ‘t Veer *et al*, 2002) and tumours with and without metastatic potential (Ramaswamy *et al*, 2003)).

Usually a test statistic or a hypothesis test (resulting in a *P*-value for each gene) is used to rank the genes with respect to their differential expression between the different tumour types or experimental conditions. Subsequently, an arbitrary threshold or rejection level *α* (genes with a *P*-value smaller than *α* are *declared* to be positive or differentially expressed) is chosen to select the genes that warrant further investigation or validation (e.g., for target discovery in drug development Gerhold *et al*, 2002).

However, due to the overlap of the *P*-values of the genes that are and are not *actually* differentially expressed (i.e., the genes whose expression is and is not affected by the difference between the experimental conditions), the choice of this rejection level has some consequences (also see Table 1

**Table 1:** Definition of true and false-positive genes (TP*_i_* and FP*_i_*) and of true and false-negative genes (TN*_i_* and FN*_i_*) at a certain level of rejection *α*=*p_i_* (*P*-value of the *i*th gene after ranking them in ascending order by *P*-value) (for each of them, the formula of the expected value is given)

	**Actually differentially expressed?**		
**Declared differentially expressed?**	**Yes**	**No**	
Yes (*P*⩽*p*_*i*_)	TP_*i*_≈ *i*−*p*_*i *_*N*_0_	FP_*i*_≈*p*_*i *_*N*_0_ (Type I error)	Pos_*i*_=*i*
No (*P*>*p*_*i*_)	FN_*i*_≈ *N*_1_−*i*+*p*_*i *_*N*_0_ (Type II error)	TN_*i*_≈(1−*p*_*i*_)*N*_0_	Neg_*i*_=*N*−*i*
			
	*N*_1_	*N*_0_	

*N*=total number of genes; *N*_0_=number of genes without actual differential expression; *N*_1_=number of genes with actual differential expression (*N*=*N*_0_+*N*_1_); Pos*_i_*=number of genes declared positive or differentially expressed at rejection level *p_i_*; Neg*_i_*=number of genes declared negative at rejection level *p_i_*.

). Firstly, genes without actual differential expression can accidentally have a *P*-value that is lower than the rejection level. Therefore, these genes are wrongfully declared to be differentially expressed. In statistics, this is also called a Type I error. This results in a number of false-positive genes that will not yield any results in further investigations. Since the number of genes in a microarray, that is not actually differentially expressed, usually is high, the number of false-positive genes at commonly used rejection levels (e.g., 5%) can be considerable (problem of multiple testing).

Secondly, the choice of the rejection level can also result in a certain number of false-negative genes (Type II error). These are the genes that are actually differentially expressed but that have a *P*-value that is larger than the rejection level, resulting in discarding potentially valid targets.

Recently, much attention has been paid in literature to the control of the number of false positives or Type I error (Keselman *et al*, 2002; Reiner *et al*, 2003; Storey and Tibshirani, 2003). Classically, by applying a Bonferroni correction, one can control the family-wise error (FWE; probability of having one or more false positives) at a given level, fixed beforehand. However, a Bonferroni correction results in extremely low rejection levels for microarray data. Controlling the FWE is therefore too stringent in this setting and results in an unacceptable Type II error (leading to an unacceptable loss of statistical power). Controlling the false discovery rate (FDR; expected fraction of genes falsely declared positive among all the genes declared differentially expressed) (Benjamini and Hochberg, 1995; Reiner *et al*, 2003; Storey and Tibshirani, 2003) is less stringent and seems a more sensible approach for microarray data but still does not control the Type II error, which could still be large and lead to the loss of a considerable number of missed targets. Control of the Type I error in microarray data often goes at the expense of the Type II error that remains uncontrolled and (too) large.

While the study of multiple testing finds its roots in genetic studies where the number of positives is usually small and control of false positives is paramount, the number of positives in studies of differential expression between patient biopsies is large and false negatives become an equally important issue. Owing to this historical reason, we believe that the control of false negatives in multiple testing methods has been somewhat overlooked.

In this paper, we present a method based on receiver-operating characteristic (ROC) curves that does not control the Type I or Type II errors but that tries to *balance* them. We aim to obtain a sensible or optimal – according to a certain criterion – trade-off between false positives and negatives. Moreover, the use of ROC curves enables us to estimate the degree of overlap between the *P*-values of genes that are and are not actually differentially expressed. This amount of overlap in its turn determines the relationship between the false positives and negatives and the level at which the optimal trade-off or balance between them can be reached (i.e., the lower the amount of overlap, the better the optimum balance). The assessment of the amount of overlap between the *P*-values by ROC curves can therefore be used to assign a quality measure to a specific microarray data set. Using two publicly available data sets (both dealing with acute leukaemia), we show that this quality measure can be used to compare different microarray data sets with respect to their ability to discriminate between genes whose expression is and is not affected by the different conditions. Since, in the near future, microarray data sets that address similar hypotheses will become increasingly available (even to such an extent that meta-analysis techniques will be necessary to analyse them simultaneously; see Rhodes *et al* (2002) and Moreau *et al* (2003)), assessing their quality could become an important issue.

## MATERIALS AND METHODS

Consider microarray data containing several sets of experiments, each analysing tissues originating from a specific group of malignancies or a specific condition, and containing expression levels for *N* genes. Assume that we have already used a certain hypothesis test to calculate the *P*-values *p*_*i*_ of the respective genes. These *P*-values reflect the probability that an equally good or better test statistic, quantifying the difference between the gene expression levels of the different conditions, is generated if a certain null hypothesis is true. In general, the null hypothesis states that there is no actual differential expression. Also assume that the genes are ordered according to this *P*-value, so that *p*_1_<*p*_2_<…<*p*_*N*_. Note that, in this paper, we chose the Wilcoxon rank sum test (a nonparametric test that examines the null hypothesis that the medians of the expression levels from *two* conditions for a certain gene are identical) to generate the *P*-values (Pagano and Gauvreau, 2000; Troyanskaya *et al*, 2002). Note, that, in principle, every procedure (e.g., through random column permutations of the data; Tusher *et al*, 2001) or hypothesis test (e.g., Kruskal–Wallis test if there are more than two conditions), that generates *P*-values for every individual gene, is suitable as long as its underlying assumptions are checked or assumed.

Starting with the estimation of the total number of genes that are and are not actually differentially expressed, we proceed by calculating the number of true positives (TP), true negatives (TN), false positives (FP), and false negatives (FN) at each rejection level. Using these estimates, the sensitivities and specificities at each rejection level can be calculated. Finally, we use these quantities to construct a ROC curve.

### Calculation of the number of genes that are and are not actually differentially expressed

Call *N*_0_ the number of genes that are not actually differentially expressed (i.e., for which the null hypothesis is true) and *N*_1_ the number of genes that are actually differentially expressed (i.e., for which the null hypothesis is false) – also see Table 1. Of course, these numbers are not known in advance and have to be estimated from the data. We use a recently introduced method (Storey and Tibshirani, 2003) for the estimation of *N*_0_, which essentially consists of an evaluation of the following formula:


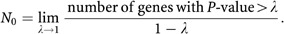


After *N*_0_ is derived, *N*_1_ can easily be estimated by *N*−*N*_0_.

To test whether this approach results in reliable estimates for *N*_0_ and *N*_1_, we applied this method on five synthetic data sets generated by the model introduced by Rocke and Durbin (2001) (also see Wang and Ethier, 2004) with increasing values for the standard deviations of the additive and multiplicative errors. These data sets contained 100 experiments each and were designed such that the values for *N*_1_ and *N*_0_ were known beforehand. For each data set, 8000 (*N*_0_) genes had a constant true expression level over the 100 experiments, while 2000 (*N*_1_) genes had a different true expression level in the first 50 experiments compared with the last 50 experiments. The five estimates of *N*_1_ varied between 1939 and 1866, dependent on the settings for the additive and multiplicative error. Note that using a two-sample *t*-test instead of the Wilcoxon test did not result in a significantly different result (estimates varied between 1909 and 1847).

### Estimation of the number of true positive, true negative, false-positive and false-negative genes

Suppose that we declare the genes with a *P*-value smaller than or equal to a certain rejection level *α*=*p*_*i*_ (*P*-value of the *i*th gene) as differentially expressed (i.e., the null hypotheses for these genes are rejected) and the genes with a *P*-value larger than this rejection level as not differentially expressed (i.e., the null hypotheses for these genes are not rejected). When the declared status of differential expression is compared with the actual status, four categories of genes (true positive, true negative, false-positive and false-negative genes) emerge that are defined in Table 1. Using the value of *N*_1_ and *N*_0_, derived in the previous section, we can calculate the number of genes in each category using the formulas from Table 1.

### Sensitivity and specificity

Using the values calculated in the previous section, the sensitivity at a certain rejection level *α*=*p*_*i*_ is defined as (Pagano and Gauvreau, 2000)


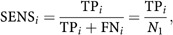


which is the fraction of actually differentially expressed genes that are declared differentially expressed. 1−sensitivity equals the probability that a gene with actual differential expression is not declared differentially expressed, which is exactly the probability of a Type II error.

The specificity at a certain rejection level *α*=*p*_*i*_ is defined as (Pagano and Gauvreau, 2000)


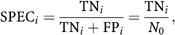


which is the fraction of genes without actual differential expression that are not declared differentially expressed. 1−specificity equals the probability that a gene without actual differential expression is declared differentially expressed, which is exactly the probability of a Type I error.

### Construction and interpretation of the ROC curve

Suppose that we calculate the sensitivities and specificities at *all* possible rejection levels *α*=*p*_*i*_ (*i*=1,…,*N*) and that we construct a graph where sensitivity is plotted *vs* 1−specificity. This graph is called the ROC curve (Dawson-Saunders and Trapp, 1994; Swets, 1996). ROC curves are a popular method to compare and characterise the performance of diagnostic tests in medicine (e.g., Epstein *et al*, 2002). They can be used here to quantify our ability to discriminate between genes with and without actual differential expression.

First of all, an ROC curve shows the trade-off between specificity and sensitivity (and hence between the Type I and Type II errors) and therefore allows for the selection of a rejection level *α*^opt^ with an optimal balance between specificity and sensitivity or between the Type I and Type II errors. Optimal can be defined in several ways and depends on the context or the requirements of the application. Often, the point on the ROC curve (and associated rejection level) with a tangent line with slope 1 is chosen, for which it can be proven that it maximises the sum of the sensitivity and specificity (and hence minimises the sum of the probability of the Type I and Type II errors) – this is also the definition of optimal that will be used in this paper. Alternatively, one can also try to optimise a more custom-defined cost function of the Type I and Type II errors that meets some specific requirements. One could, for example, use a cost function that puts more weight on either the Type I or Type II error (see the example in the Supplementary online material), depending on which is most important or practical for a specific situation or researcher (it is, therefore, difficult to give general or objective guidelines for the values of these weights). In fact, by minimising the sum of the probability of the Type I and Type II errors (as said, this is done in this paper), the number of false positives and negatives are weighed by the inverse of the number of genes without and with actual differential expression, respectively. This means, for example, that the ‘cost’ of a false negative will be higher if the number of genes that are actually differentially expressed (or that are actually positive) is lower and vice versa, which is logical since the impact of missing a rare target is higher than the impact of missing one of many targets.

Secondly, the area under the ROC curve (AUC) has a special meaning (see Hanley and McNeil (1982) for a method to calculate the AUC and its standard deviation). Suppose we randomly select a gene *g*_*i*_ with actual differential expression with *P*-value *p*_*i*_ and a gene *g*_*j*_ without actual differential expression with *P*-value *p*_*j*_, then it can be proven that


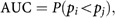


that is, the AUC equals the probability that the *P*-value of the gene with actual differential expression is lower than the *P*-value of the gene without actual differential expression and therefore it is the probability that *p*_*i*_ and *p*_*j*_ are ranked correctly. The AUC quantifies how well the genes whose expression is and is not affected by the difference between the tumour types can be discriminated using the *P*-values of these genes. The AUC increases if the overlap between the *P*-values of the genes with and without actual differential expression decreases. This means that the level of the optimal balance between Type I and Type II (e.g., the maximum of the sum of the specificity and sensitivity) increases if the AUC increases. Therefore, the AUC can be seen as a quality measure with respect to the detection of differential expression for a specific set of microarray experiments. Provided the same hypothesis test is consistently applied, the AUC can be used to compare (see Hanley and McNeil (1983) for a method to compare AUCs) the ability of different gene expression data sets to discriminate between genes whose expression is and is not affected by the difference in conditions. For example, one could calculate this quality measure for several data sets, which study gene expression levels under the same conditions, from different sources or institutions. As another example, one could try to study the effect on the differential expression and on this quality measure by a change in one or both conditions (see Results section).

### Data sets

We applied the methodology described above on microarray data originating from two sources that contain measurements from two or three classes of acute leukaemia.

The first data set (Golub *et al*, 1999) studies the expression profiles of bone marrow or peripheral blood of 72 patients with acute lymphoblastic (ALL; Condition 1; 47 patients) or myeloid leukaemia (AML; Condition 2; 25 patients) using an Affymetrix chip. In the original publication the patients are divided into training and a test set, but this distinction is not important here. Although the separation between the two conditions is more pronounced than in most other cases, this data set can still be considered as a benchmark (paper cited over 1090 times). The data contains *N*=7129 genes and can be downloaded from http://www.genome.wi.mit.edu/c ancer/.

The second data set (Armstrong *et al*, 2002) also contains several microarray experiments obtained from patients with ALL or AML and from a third class or condition containing ALLs with an MLL translocation (called MLL leukaemia). Armstrong *et al* discovered that MLL leukaemias have a distinct expression pattern and can be considered as a separate disease distinguishable from ALL and AML. The data set they used is publicly available and can be downloaded from http://research.dfci.harvard.e du/korsmeyer/Supp_pubs/Supp_Ar mstrong_Main.html. It contains expression profiles for 12 582 genes measured using Affymetrix technology. In total, 24 ALL patients, 28 AML patients and 20 MLL patients are available. This resulted in a data set containing 72 patients.

## RESULTS

### Comparison of data from different sources studying the same conditions

We analysed the data from Golub *et al* and Armstrong *et al* separately with respect to the detection of differential expression between ALL and AML (for this analysis, we removed the 20 MLL patients from Armstrong *et al*). The results can be inspected and compared in Table 2

**Table 2:** Results for the data from Golub *et al* (detection of differential expression between ALL and AML) and from Armstrong *et al* (detection of differential expression between ALL and AML, between ALL and MLL and between MLL and AML)

	**Golub *et al* (ALL-AML)**	**Armstrong *et al* (ALL-AML)**	**Armstrong *et al* (ALL-MLL)**	**Armstrong *et al* (MLL-AML)**
*N*	7129	12 582	12 582	12 582
*N*_0_	3876	3084	8119	4527
*N*_1_	3253	9498	4463	8055
AUC (%) (95% CI)	91.39 (90.68–92.10)	95.13 (94.78–95.48)	85.98 (85.24–86.72)	94.83 (94.46–95.20)
*α*^opt^	0.18 (=*p*_3429_)	0.11 (=*p*_8633_)	0.22 (=*p*_5193_)	0.13 (=*p*_7589_)
SENS^opt^ (%)	84.03	87.26	76.75	86.97
SPEC^opt^ (%)	82.06	88.56	77.97	86.78
SENS^opt^+SPEC^opt^ (%)	166.09	175.82	154.71	173.76

**Figure 1:**
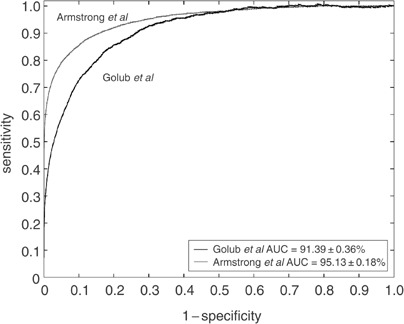
Receiver-operating characteristic curves for the data from Golub *et al* and from Armstrong *et al* with respect to the detection of differential expression between ALL and AML.

*N*=total number of genes; *N*_0_=number of genes without actual differential expression; *N*_1_=number of genes with actual differential expression; AUC=area under the ROC curve; *α*^opt^=rejection level where the optimal balance between specificity and sensitivity is reached (i.e., the rejection level that maximises the sum of sensitivity and specificity – for the first two columns, these are also the rejection levels associated with the points on the ROC curves in Figure 1 with tangent lines with slope 1); SENS^opt^=sensitivity at *α*^opt^; SPEC^opt^=specificity at *α*^opt^.

and Figure 1

. The AUC of the data from Armstrong *et al* (95.13%) is significantly (*P*<0.0001; two-sided, unpaired test; Hanley and McNeil, 1983) different from the AUC derived from the data set from Golub *et al* (91.39%), which is reflected in the fact that the level of the optimal balance between (or, in our case, the maximum sum of) sensitivity and specificity is higher in the data from Armstrong *et al* when compared to the data from Golub *et al* (175.82 *vs* 166.09%).

Note that in the Supplementary online material accompanying this paper, the results of a similar analysis studying differential expression between human breast tumours that are moderately and poorly differentiated can be inspected.

### Effect of a change in condition

We analysed the data from Armstrong *et al* with respect to the detection of differential expression between ALL and MLL (after removal of the 28 AML patients) and with respect to the detection of differential expression between MLL and AML (after removal of the 24 ALL patients) and compared this with the results from the previous section with respect to the detection of differential expression between ALL and AML on the same data set. The results can also be inspected in Table 2. The difference between MLL and AML did not result in any statistically significant change in AUC when compared with the difference between ALL and AML. However, the difference between ALL and MLL did result in a significant decrease in AUC when compared with the difference between ALL and AML (85.98 *vs* 95.13%, *P*<0.0001), which also resulted in a considerable decrease in the level of the optimal balance between sensitivity and specificity (maximum of sensitivity+specificity=154.71 *vs* 175.82%), as could be expected.

## DISCUSSION

In this paper, we describe and use a procedure for the detection of differential expression based on the construction of ROC curves. In contrast with current practice only to control the Type I error, this method enables to balance the Type I and Type II errors according to a certain criterion or cost function and enables, through the AUC, to quantify our ability to discriminate between genes with and without actual differential expression in a specific data set and using a certain hypothesis test. As was shown in the Results section, the AUC also reflects how well the Type I and Type II errors can be balanced. We therefore propose to use the AUC as a quality measure to compare data sets for their appropriateness to detect differential expression, provided the same hypothesis test is used consistently.

In theory, using other tests to derive the *P*-values could have an effect on the result of our analysis. Therefore, as an example, we repeated all the analyses described in this manuscript using a two-sample (parametric) *t*-test (although we could be far from certain that its distributional assumptions were satisfied) instead of the Wilcoxon test. This did not significantly change the estimated values for *N*_1_. The resulting AUCs, however, differed somewhat from the values observed in Table 2, but their ranking did not change, giving exactly the same conclusions for our comparisons.

The quality measure proposed here could be used for different types of comparisons, of which we illustrated two. As a first example, we investigated how this quality measure could be used to compare data sets that study the same conditions (in this case ALL and AML) but that originate from different sources. After comparing their AUCs, we concluded that the data from Armstrong *et al* are more appropriate to discriminate between genes that are and are not differentially expressed than the data from Golub *et al*, although the last data set contained more experiments than the first (72 *vs* 52). In our opinion, the optimisation of the Affymetrix technology and protocol (year 2002 *vs* 1999) and perhaps a more optimal selection of the genes arrayed on the chip for Armstrong *et al* could have contributed to this difference in quality, which was accurately detected by the rise in AUC. The methodology described here could be suited to compare the performance of different microarray platforms (e.g., cDNA microarrays *vs* Affymetrix). It might also be a good idea to use our procedure to detect whether there are differences in quality between different data sets performed on an equal number of samples captured on the same platform or chip set.

As a second example, we examined what the effect on the AUC could be of a change in condition (replacement of ALL or AML patients by MLL patients). The difference between MLL and AML did not result in a significant decrease in AUC when compared to the difference between ALL and AML, while the difference between ALL and MLL did. The lower number of experiments that were available for the analysis of the difference between ALL and MLL (44 *vs* 52 for the analysis of the difference between ALL and AML) could have partially caused the significant drop in AUC, but this was, to a lesser extent, also true for the analysis of the difference between MLL and AML (48 patients), which did not show a drop in AUC. The behaviour of the AUC and the results in Table 2 suggest that the degree of differential expression between ALL and MLL is less pronounced than the degree of differential expression between ALL and AML or between MLL and AML. This seems plausible, because the leukaemic cells in MLL patients have a lymphoblastic morphology and have previously been classified as ALL. Again, this has been accurately detected by our analysis of the AUCs.

Note that the estimated values for *N*_1_ in Table 2 are considerable, especially for the difference between ALL and AML in the data from Armstrong *et al* (9498 genes out of 12 582 are calculated to be actually differentially expressed). The fact that these estimates for *N*_1_ also include the genes whose difference in expression is only subtle (e.g., these might be the genes that do not really play a role in the difference between the tumour types and whose expression is only changed as a side effect downstream of the main biological processes responsible for the different phenotypes) could explain this. However, we cannot exclude that an experimental bias between the two conditions not removed by adequate pre-processing could partially be responsible for these observations.

The use of a cost function that minimises the sum of the probability of a Type I and Type II error results in relatively large values for *α*^opt^ (in Table 2 between 0.11 and 0.22), which in turn leads to the selection of a number of genes that is (too) high (several thousands in Table 2). This result is inherent to the use of microarray technology where a huge number of genes are analysed at once and where, in the ideal case, one should be prepared to accept a considerable number of genes that merit further investigation. We realise however that, at this moment, the validation of this large number of potential targets is difficult and that in many practical settings a rejection level lower than the optimal one has to be used. However, one should realise that the choice of such a lower rejection level can and will lead to the loss of many (or in some cases even the most important) targets. In our opinion, calculation of the optimal rejection level is still useful in this situation in order to evaluate how far we are removed from the ideal case. Moreover, and in some specific research settings, the optimal rejection level can effectively guide the selection of the genes that could be useful to be included in further investigations (for example, if one wishes to build a custom chip with a limited number of genes tailored for a specific biological or medical question). If the selection of a lower rejection level, however, is still necessary, one could, as a second choice, try to formalise this approach by the definition of a custom cost function. This is illustrated in the study of differential expression between grade 2 and 3 breast tumours presented in the Supplementary online material.

In our opinion, several other situations can be conceived where a comparison of AUCs could be informative, although we did not study them in detail in this manuscript. For example, one could compare the AUCs of a data set for which the raw experimental data have been pre-processed – in order to remove different systematic sources of experimental variation from microarray data – using different strategies (e.g., Lowess fit (Yang *et al*, 2002), ANOVA-based methods (Kerr *et al*, 2000), etc) and select a pre-processing strategy that results in a maximal AUC or maximal discrimination between the genes that are and are not differentially expressed.

Evaluation of the usefulness of additional experiments with respect to the detection of differential expression is another example where an ROC analysis could be valuable. Suppose one has performed a basic set of microarray experiments (under one of two or more conditions) and suppose one performs a set of additional experiments in order to obtain a more optimal identification of the genes that are actually differentially expressed. Comparison of the AUCs of the basic set and of the basic+additional set could quantify whether this has succeeded and could even help us to decide if more additional experiments would be beneficial (e.g., if the set of additional experiments has not resulted in a satisfactory rise in AUC, it could be expected that more additional experiments also will fail to do this).
