# Supplementary material for: Balancing false positives and false negatives for the detection of differential expression in malignancies
Source: Br J Cancer. 2004 Aug 31;91(6):1160–5. doi: 10.1038/sj.bjc.6602140 (PMC2747693; doi:10.1038/sj.bjc.6602140)
Supplement: Supplementary online material [file 91-6602140x1.doc]

# Balancing false positives and false negatives for the detection of differential expression in malignancies

**Frank De Smet, Yves Moreau, Kristof Engelen, Dirk Timmerman, Ignace Vergote and Bart De Moor**

# Supplementary online material:

# Comparison of data from different sources studying grade 2 and grade 3 breast tumours

In this online supplement we present the results of an additional comparison we performed using our procedure. We compared two microarray data sets that study human breast tumours that are moderately or poorly differentiated (grade 2 or 3 - the degree of differentiation reflects the degree of anaplasia or the degree of malignancy of the tumour and is an important prognostic factor).

The first data set was published by Perou et al. (2000). They analysed surgical specimens of human breast tumours using cDNA technology with a common reference sample. Their study contained, among others, 37 tumours that were moderately or poorly differentiated (grade 2 or 3 – the degree of differentiation reflects the degree of anaplasia or the degree of malignancy of the tumour and is an important prognostic factor). Twenty of these tumours were sampled twice (before and after a 16-week course of doxorubicin chemotherapy or paired with a lymph node metastasis) resulting in 57 microarray experiments (21 with grade 2 and 36 with grade 3). The raw data for each experiment (9216 genes) and the associated grade were downloaded from <http://genome-www.stanford.edu/molecularportraits/>. We calculated the ratio of the difference between the total and background intensity from the tumour and reference sample. Subsequently, a simple normalization was performed by multiplying each array with a single scaling factor so that the median ratio on each array was 1 (Alizadeh et al., 2000). Finally, a logarithmic transformation (base 2) was performed.

van ‘t Veer et al. (2002) also studied primary breast tumours (from sporadic lymph node negative patients that did and did not develop distant metastases within five years and from patients with BRCA1 or BRCA2 germline mutations) using a cDNA microarray. In total 117 patients were analysed but only 105 of them had a tumour with grade 2 (27 patients) or grade 3 (78 patients). We downloaded the log-ratios from the different experiments on <http://www.rii.com/publications/default.htm> (24481 genes) and the data about the degree of differentiation from the supplementary information on [http://www.nature.com](http://www.nature.com/).

The results of our comparison can be inspected in Table 3 and Figure 2. For the study of Perou et al. and with respect to the detection of differential expression between grade 2 and grade 3, the AUC was 87.99%. For the study of van ‘t Veer et al., the AUC was 90.54%, which was significantly different (p = 0.0001) from the AUC from Perou et al. From this we can conclude that the study of van ‘t Veer et al., when compared to the study of Perou et al., is more appropriate to study differential gene expression between breast tumours with grade 2 and 3. Again, possible causes that could have attributed to this gap in quality are differences in technology, differences in experimental protocol and experimental setup, differences in surgical procedure and quality of the resected tumour biopsy, the choice of the genes on the array (more specifically chosen to study breast cancer in van ‘t Veer et al.) and so on. Since the determination of the degree of differentiation can vary between pathologists, this is also a factor that could have contributed. Note that the value for N_1_ is considerable (especially in the study of van ‘t Veer), suggesting that tumour cells with a different grade have a profoundly different phenotype.

In order to determine the rejection level α^opt^ where the optimal balance between Type I and Type II errors is reached and contrary to the definition of optimal that was given in the manuscript, we minimized, as an example, a custom cost function that puts ten times more weight on a Type I error than on a Type II error. This is equivalent with maximizing the following function: 10 × SPEC_i_ + SENS_i_. As could be expected and in general, this cost function results in values for α^opt^ (see Table 3) that are substantially lower when compared to the values that result from the definition of optimal that is used in the paper (where the sum of the sensitivity and specificity is maximized – compare with the values for α^opt^ in Table 2 in the paper). Again, the difference in AUC between Perou et al. and van ‘t Veer et al. is reflected in the level of the optimal balance between Type I and Type II error, which is better in the study of van ‘t Veer et al. than in the study of Perou et al. (the level of the optimal balance is quantified by the value of the cost function in α^opt^: 10 × SPEC^opt^ + SENS^opt^ – see the last row of Table 3).

## References

Alizadeh, A.A., Eisen, M.B., Davis, R.E., Ma, C., Lossos, I.S., Rosenwald, A., Boldrick, J.C., Sabet, H., Tran, T., Yu, X., Powell, J.I., Yang, L., Marti, G.E., Moore, T., Hudson, J., Lu, L., Lewis, D.B., Tibshirani, R., Sherlock, G., Chan, W.C., Greiner, T.C., Weisenburger, D.D., Armitage, J.O., Warnke, R., Levy, R., Wilson, W., Grever, M.R., Byrd, J.C., Botstein, D., Brown, P.O., and Staudt, L.M. (2000) Distinct types of diffuse large B-cell lymphoma identified by gene expression profiling. *Nature*, **403**, 503-511.

Perou, C.M., Sorlie, T., Eisen, M.B., van de Rijn, M., Jeffrey, S.S., Rees, C.A., Pollack, J.R., Ross, D.T., Johnsen, H., Akslen, L.A., Fluge, O., Pergamenschikov, A., Williams, C., Zhu, S.X., Lonning, P.E., Borresen-Dale, A.L., Brown, P.O., and Botstein, D. (2000) Molecular portraits of human breast tumours. *Nature*, **406**, 747-752.

van 't Veer, L.J., Dai, H., van de Vijver, M.J., He, Y.D., Hart, A.A., Mao, M., Peterse, H.L., van der Kooy, K., Marton, M.J., Witteveen, A.T., Schreiber, G.J., Kerkhoven, R.M., Roberts, C., Linsley, P.S., Bernards, R., and Friend, S.H. (2002) Gene expression profiling predicts clinical outcome of breast cancer. *Nature*, **415**, 530-536.

**Table 3.** Results of our analysis for the data from Perou et al. and van ‘t Veer et al. with respect to the detection of differential expression between grade 2 and 3 breast tumours.

|  | Perou et al. | van ‘t Veer et al. |
| --- | --- | --- |
| N | 9216 | 24481 |
| N_0_ | 7910 | 14273 |
| N_1_ | 1306 | 10208 |
| AUC (%) [95% CI] | 87.99 [86.76-89.22] | 90.54 [90.12-90.96] |
| α^opt^ | 0.01 (=p_295_) | 0.01 (=p_3919_) |
| SENS^opt^ (%) | 16.01 | 36.56 |
| SPEC^opt^ (%) | 98.91 | 98.66 |
| SENS^opt^ + 10 × SPEC^opt^ (%) | 1005.15 | 1023.18 |

N = total number of genes; N_0_ = number of genes without actual differential expression; N_1_ = number of genes with actual differential expression; AUC = area under the ROC curve; α^opt^ = rejection level where the optimal balance between specificity and sensitivity is reached (in this case the rejection level that maximizes the sum of sensitivity and ten times the specificity); SENS^opt^ = sensitivity at α^opt^; SPEC^opt^ = specificity at α^opt^.


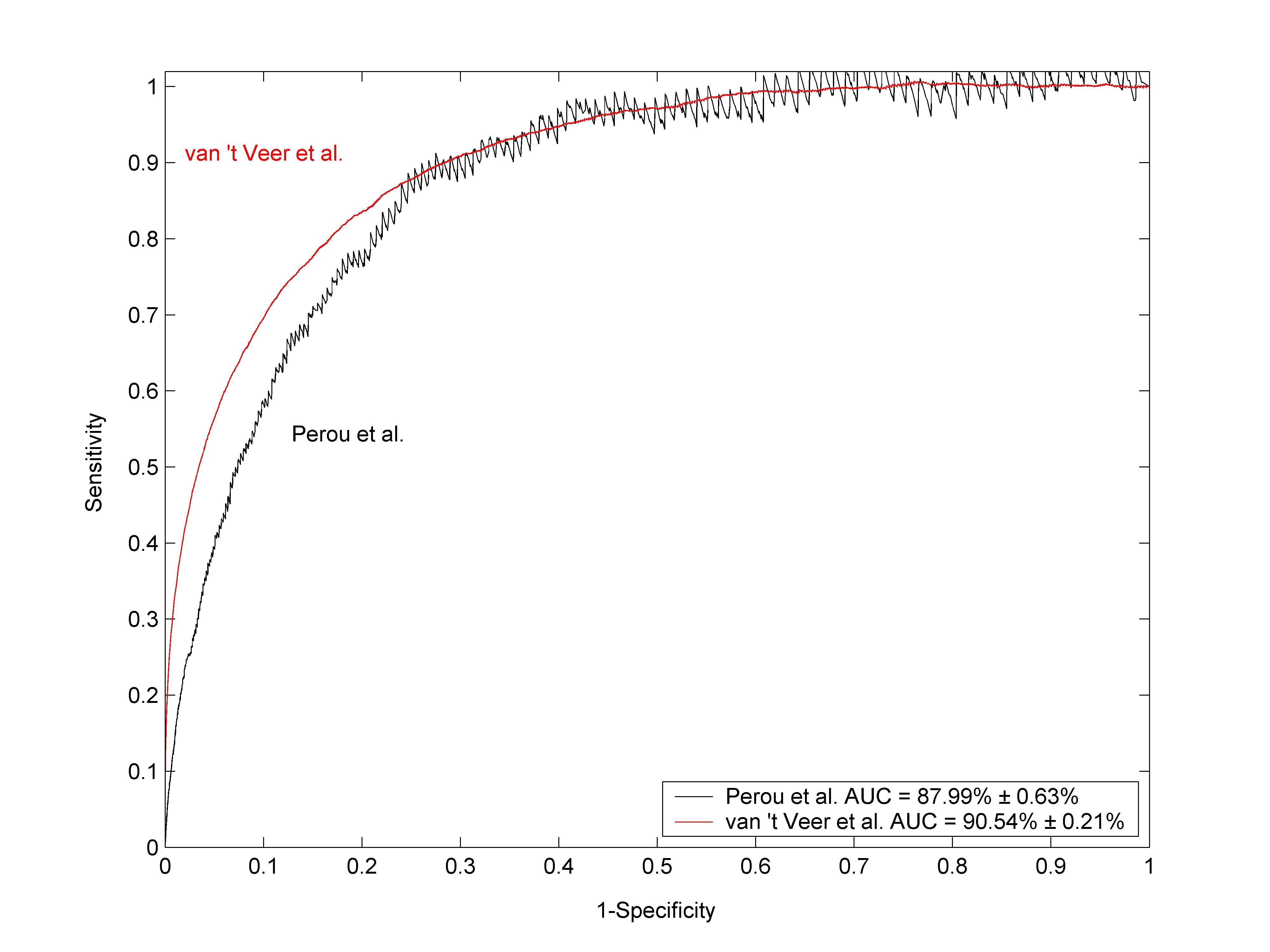
**Fig 2.**  ROC curves for the data from Perou et al. and from van ‘t Veer et al. with respect to the detection of differential expression between grade 2 and 3 breast tumours.
